# Exercise Capacity and Response to Training Quantitative Trait Loci in a NZW X 129S1 Intercross and Combined Cross Analysis of Inbred Mouse Strains

**DOI:** 10.1371/journal.pone.0145741

**Published:** 2015-12-28

**Authors:** Michael P. Massett, Joshua J. Avila, Seung Kyum Kim

**Affiliations:** Department of Health and Kinesiology, Texas A&M University, College Station, Texas, United States of America; West Virginia University School of Medicine, UNITED STATES

## Abstract

Genetic factors determining exercise capacity and the magnitude of the response to exercise training are poorly understood. The aim of this study was to identify quantitative trait loci (QTL) associated with exercise training in mice. Based on marked differences in training responses in inbred NZW (-0.65 ± 1.73 min) and 129S1 (6.18 ± 3.81 min) mice, a reciprocal intercross breeding scheme was used to generate 285 F_2_ mice. All F_2_ mice completed an exercise performance test before and after a 4-week treadmill running program, resulting in an increase in exercise capacity of 1.54 ± 3.69 min (range = -10 to +12 min). Genome-wide linkage scans were performed for pre-training, post-training, and change in run time. For pre-training exercise time, suggestive QTL were identified on Chromosomes 5 (57.4 cM, 2.5 LOD) and 6 (47.8 cM, 2.9 LOD). A significant QTL for post-training exercise capacity was identified on Chromosome 5 (43.4 cM, 4.1 LOD) and a suggestive QTL on Chromosomes 1 (55.7 cM, 2.3 LOD) and 8 (66.1 cM, 2.2 LOD). A suggestive QTL for the change in run time was identified on Chromosome 6 (37.8 cM, 2.7 LOD). To identify shared QTL, this data set was combined with data from a previous F_2_ cross between B6 and FVB strains. In the combined cross analysis, significant novel QTL for pre-training exercise time and change in exercise time were identified on Chromosome 12 (54.0 cM, 3.6 LOD) and Chromosome 6 (28.0 cM, 3.7 LOD), respectively. Collectively, these data suggest that combined cross analysis can be used to identify novel QTL and narrow the confidence interval of QTL for exercise capacity and responses to training. Furthermore, these data support the use of larger and more diverse mapping populations to identify the genetic basis for exercise capacity and responses to training.

## Introduction

Cardiorespiratory fitness or exercise capacity determined by a graded treadmill test is an independent predictor of cardiovascular disease and all-cause mortality in men and women [1–3]. Improving cardiorespiratory fitness through increased physical activity can significantly reduce the risk of all-cause mortality [1, 4], regardless of the level of initial fitness [5]. Although regular exercise is recommended for optimal health, adherence to a standardized exercise-training program does not guarantee improvements in fitness. On the contrary, responses to exercise training are highly variable such that some individuals can show minimal or no improvements in exercise capacity, i.e., cardiorespiratory fitness [6–11]. Evidence from linkage analysis and genome-wide association studies indicates that genetics contribute significantly to individual variation in both baseline exercise capacity and the response to training [12–15]. Linkage studies have identified several genomic markers linked to training-induced changes in oxygen consumption and maximal power output [12, 13]. In addition, single nucleotide polymorphisms (SNPs) and skeletal muscle transcripts associated with changes in oxygen consumption in response to exercise training have been identified using genome-wide approaches [14, 15]. However, despite these successes, much of the underlying genetic basis of exercise capacity and responses to exercise training remain to be elucidated [16].

As an alternative to exercise intervention trials in humans, mice and rats are being utilized to identify the genetic basis for variation in exercise capacity and training responses. Based on significant strain differences in baseline or pre-training exercise capacity measured during a graded treadmill test, quantitative trait loci (QTL) for exercise capacity have been identified in rats and mice [17–21]. In addition, a genome wide linkage scan for exercise capacity and responses to training was performed using in an F_2_ population derived from inbred FVB/NJ (FVB) and C57BL/6J (B6) mice [20]. In (FVB x B6) F_2_ population, several significant and suggestive QTL for pre- and post-training exercise capacity and the responses to training (i.e., change in exercise capacity) were identified. However, that study was conducted on a relatively small population (< 200) of F_2_ mice and mapping resolution was limited by the variation present in the genomes of the two mouse strains. Therefore, many of the QTL intervals were relatively large, making candidate gene identification difficult.

To increase the power and mapping resolution of traditional linkage studies, Li et al. developed a method for combing data from multiple F_2_ crosses [22]. This combined cross analysis has been utilized to increase the resolution of shared QTL and identify new QTL not identified in individual crosses for traits such as bone mineral density, encephalomyelitis, wound healing, and plasma lipids [22–27]. Due to the limited data available for exercise capacity and responses to training, this approach has not been utilized for exercise-related traits in mice. Therefore, the purpose of this study is two-fold: first, to map genetic loci that regulate exercise capacity and responses to training in a new independent F_2_ cross, and second, to perform a combined cross analysis using this new data and the results of a previously published F_2_ intercross.

## Materials and Methods

### Animals

All procedures adhered to the established National Institutes of Health guidelines for the care and use of laboratory animals and were approved by the Institutional Animal Care and Use Committee at Texas A&M University. All mice were housed in standard caging and allowed food (Standardized Laboratory Rodent Diet) and water *ad libitum* and maintained at an ambient temperature of 22–24°C on a 12 hr light:dark schedule. Seven-week old male and female 129S1/SvImJ and NZW/LacJ were purchased from Jackson Laboratory (Bar Harbor, ME), allowed to acclimate for one week, and then screened for exercise capacity and changes in exercise capacity in response to training. Additional male and female mice from each strain were mated to generate reciprocal F_1_ offspring. F_1_ offspring were then intercrossed to generate 285 F_2_ mice (130 female, 155 male). At approximately 8 weeks of age (59–64 days old), F_2_ mice were screened for exercise capacity and completed the exercise training program as described below.

### Exercise Performance Test

Eight-week old mice completed a graded treadmill run to exhaustion on a motorized rodent treadmill with an electric grid at the rear of the treadmill (Columbus Instruments, Columbus, OH) after 2 days of familiarization as previously described [19, 20]. For the exercise test, mice performed a 9 minute warm-up by walking on the treadmill at 9 m/min and 0° grade. Speed was then increased by 2.5 m/min every 3 minutes from a starting speed of 10 m/min. The incline progressively increased 5° every 9 min to a maximum of 15°. The test continued until each mouse was unable to maintain running speed despite repeated contact with the electric grid. At this point, running time (in min) was recorded and each mouse was immediately removed from the treadmill and returned to its home cage. Each mouse performed two exercise tests separated by 48 hours and the average test duration was used as a measure of maximal exercise capacity. Exercise performance tests were repeated after the 4-week exercise-training program was completed.

### Exercise Training

The exercise-training program was designed to match those previously reported by our laboratory [20, 28]. This protocol and similar protocols have been shown to produce cardiovascular and skeletal muscle adaptations [20, 28–32]. For exercise training, all F_2_ mice ran on a six-lane treadmill 5 days/week, 60 min/day for 4 weeks at a final intensity of 16.5 m/min up a 10° incline. This workload is approximately 65% of the maximal workload attained during the pre-training exercise performance test. F_2_ mice were trained in 6 cohorts of approximately 48 mice each. A minimal number of sedentary F_2_ mice were utilized as time controls. These mice were exposed to the treadmill but not made to run and showed no changes in exercise performance.

### Genotyping

DNA was extracted from tail samples and genotyping performed using competitive allele specific PCR SNP genotyping system (KBiosciences, Herts, UK) [33, 34]. Mice were genotyped using 138 single nucleotide polymorphism (SNPs) markers spaced at 12 cM interval [33, 34]. For the previously published intercross between C57BL/6J (B6) and FVB/NJ (FVB) mice, F_2_ mice were genotyped at 104 markers spaced at approximately 20 cM intervals [20]. Subsequent genotyping was performed on these F_2_ mice to increase the total to 142 SNPs with 12.6 cM spacing.

### QTL Analysis

QTL mapping was performed using R/qtl [35]. For each exercise phenotype, one-dimensional genome scans were performed with no covariates included and with sex as an additive and interacting covariate. If significant QTL were identified using sex as a covariate, defined as a difference in LOD scores (ΔLOD) ≥ 2.0 between scans that included additive and interacting covariates, male and female mice were analyzed separately [36]. Permutation tests (~1,000 repetitions) were used to calculate experiment-specific threshold values for LOD scores and determine the significance of linkage between marker genotype and phenotype [37]. LOD scores surpassing the P < 0.05 threshold were considered significant and those surpassing the P < 0.63 were considered suggestive. Bayesian credible interval function was used to estimate QTL confidence intervals [38]. Multiple regression analysis was used to determine the contribution of each QTL to each exercise phenotype.

### Combined cross analysis

To increase mapping power and improve resolution of QTL intervals, a combined cross analysis was performed using data from the current cross (NZW x 129S1) and the previously published FVB x B6 intercross [20]. The means and variances for the exercise phenotype data were significantly different between crosses; therefore all phenotype data were converted to z-scores to stabilize the variances [22]. Z-score conversions were performed for each individual cross before using in the combined cross analysis. Genotype data for both crosses were re-coded to “HH” for mice homozygous for the allele contributing to the high exercise phenotype, “LL” for mice homozygous for the allele contributing to the low exercise phenotype, and “LH” for mice with heterozygous alleles [22]. Combined cross analysis was performed for each exercise phenotype using “cross” and “sex” as additive covariates.

### Statistical Analysis

Values are expressed as mean ± SE. A two-way analysis of variance (ANOVA, sex and strain) was used for comparisons among inbred parental strains and the (NZW x 129S1) F_2_ population. A one-way ANOVA followed by Tukey’s post-hoc test was used for comparisons across populations within each sex and across genotypes for each peak SNP marker. A Student’s t-test was used to compare phenotypes between males and females within each population. Pre- vs. post training comparisons within a population were made using paired student’s t-tests. Statistical significance was set at P < 0.05.

## Results

### Phenotypes in parental strains

Body mass and exercise phenotypes are shown in Table 1. Pre-training body mass was significantly greater in NZW mice (25.4 ± 0.9 g, n = 12, P < 0.001) compared with 129S1 mice (18.7 ± 0.7 g, n = 17). Post-training body mass also was significantly greater in NZW mice (28.2 ± 0.9 g, P < 0.001) compared with 129S1 mice (20.9 ± 0.7 g). Body mass significantly increased over time in both strains (129S1: P = 0.0003; NZW: P < 0.0001); however, the change in body mass was similar between the strains (NZW: 2.8 ± 0.4 g; 129S1: 2.2 ± 0.5 g, P = 0.8). For both strains, body mass was lower in females than in males at each time point (Table 1). Exercise capacity also showed significant strain effects. Pre-training exercise time (NZW: 27.4 ± 0.6 min; 129S1: 29.2 ± 1.1 min, P = 0.008), post-training exercise time (NZW: 26.8 ± 0.8 min; 129S1: 35.3 ± 1.8 min, P < 0.0001), and the change in exercise time (NZW: -0.7 ± 0.5 min; 129S1: 6.2 ± 0.9 min, P < 0.0001) were significantly greater in 129S1 mice compared with NZW mice. For 129S1 mice, post-training time was significantly greater than pre-training time (P < 0.0001), indicating a positive response to exercise training (Table 1). Conversely, there was no difference between pre- and post-training exercise times in NZW mice (P = 0.23), inferring that they did not respond to training. Within each strain, sex differences were also observed. In 129S1, male mice ran significantly longer pre-training and post-training compared with females (Table 1). The response to training was greater in males as well. For NZW, female mice had greater pre-and post-training exercise times compared with males. No difference between males and females was observed for the response to training.

**Table 1:** Body mass and exercise time in inbred parental and F_2_ mice.

			Body Mass, g			Time, min		
Group	Sex	N	Pre ^a^ ^,^ ^b^	Post ^a^ ^,^ ^b^	Delta ^b^	Pre ^b^ ^,^ ^c^	Post ^a^ ^,^ ^b^ ^,^ ^c^	Delta ^b^ ^,^ ^c^
129S1	female	11	17.2 ± 0.7*	19.2 ± 0.6* ^,^ ^†^	2.0 ± 0.7	26.0 ± 0.4*	30.3 ± 0.7* ^,^ ^†^	4.3 ± 1.0*
	male	6	21.3 ± 0.3	24.0 ± 0.3^†^	2.6 ± 0.2	35.0 ± 0.6	44.6 ± 0.4^†^	9.7 ± 0.5
NZW	female	6	22.9 ± 0.3*	25.7 ± 0.4* ^,^ ^†^	2.9 ± 0.3	29.3 ± 0.3*	28.7 ± 0.5*	-0.7 ± 0.4
	male	6	27.9 ± 0.8	30.6 ± 0.8^†^	2.7 ± 0.8	25.5 ± 0.3	24.9 ± 1.1	-0.7 ± 1.0
F_2_	female	130	25.1 ± 0.3*	25.9 ± 0.3* ^,^ ^†^	0.8 ± 0.1*	30.0 ± 0.3*	31.8 ± 0.2* ^,^ ^†^	1.9 ± 0.3
	male	155	30.7 ± 0.3	32.2 ± 0.3^†^	1.6 ± 0.2	29.1 ± 0.2	30.4 ± 0.3^†^	1.2 ± 0.3

Values are mean ± SE.

^a,^ significant effect of sex (P < 0.05);

^b,^ significant effect of strain (P < 0.05);

^c,^ significant strain by sex interaction (P < 0.05);

*, P < 0.05 compared with males from same strain;

^†,^ P < 0.05 compared with pre-training value.

### Phenotypes in F_2_ intercross

Body mass in the F_2_ population ranged from 18.2 to 50.2 g before training and 19.5 to 52.1 g after training. Post-training body mass was significantly greater than pre-training body mass (P < 0.0001). The range for the change in body mass was -6.1 to +12.8 g. Overall, pre-training body mass was significantly greater in F_2_ mice (28.1 ± 0.3 g, n = 285) than pre-training body mass in the parental 129S1 (P < 0.0001) and NZW (P = 0.03) strains. Post-training body mass was higher in F_2_ mice (29.3 ± 0.3 g) compared with 129S1 mice (P < 0.0001), but not different from NZW mice (P = 0.7). Conversely, the change in body mass was significantly smaller in F_2_ mice (1.2 ± 0.1 g) compared to NZW mice (P = 0.01), but not significantly different from 129S1 (P = 0.054). Differences between males and females were observed for each body mass phenotype in the F_2_ cohort (Table 1). Pre-training exercise time in the F_2_ cohort was 29.5 ± 0.2 min (range: 21.6 to 40.8 min). Post-training exercise time was 31.0 ± 0.2 min (range: 18.1 to 40.3 min), which is significantly greater than pre-training exercise time (P < 0.0001). Pre-training exercise time in the F_2_ cohort was similar to 129S1 (P = 0.2) and greater than NZW (P = 0.03) mice. Post-training time was significantly lower in F_2_ mice compared with 129S1 (P < 0.0001), but significantly higher than NZW (P < 0.0001) mice. The response to training in F_2_ mice was 1.5 ± 0.2 min (range: -10.1 to 12.4 min) and similar to that in NZW (P = 0.1) and significantly lower than the response to training in 129S1 (P < 0.0001) mice. In F_2_ mice, pre- and post-training exercise times were significantly greater in females compared with males. Responses to training were not different between the sexes.

### QTL analysis in NZW x 129S1 intercross mice

**Fig 1:**
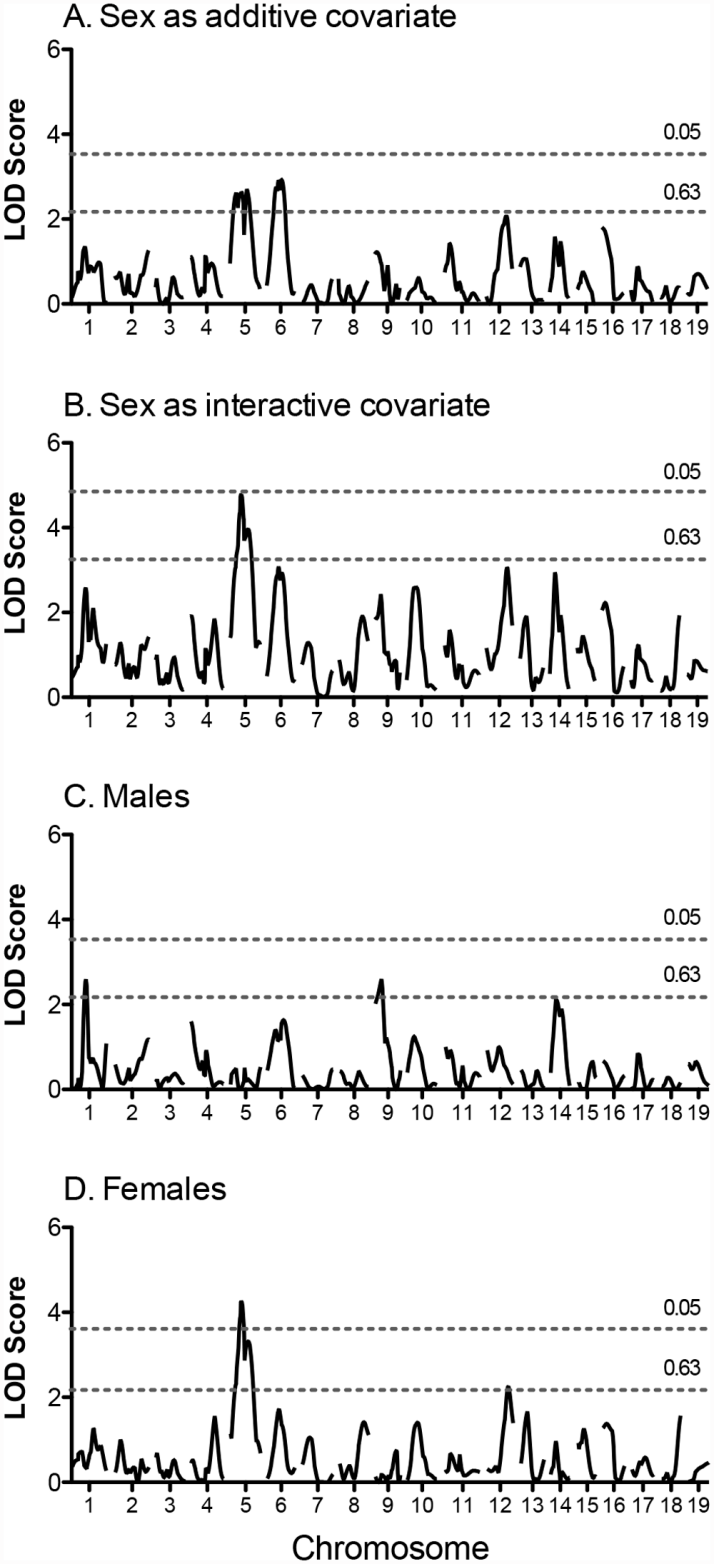
Genome-wide scan for pre-training exercise time in (NZW x 129S1) F_2_ mice. Scans were performed for the entire population with “sex” as an additive covariate (A) or interactive covariate (B), and in males (C) and females (D) separately. Horizontal lines represent significant (P = 0.05) and suggestive (P = 0.63) logarithm of odds (LOD) thresholds, respectively. LOD thresholds were determined by permutation testing using 1000 permutations.

Genome-wide linkage scans for pre-training time are shown in Fig 1. Two suggestive QTL were identified for pre-training exercise time on Chromosomes 5 and 6 when sex was included as an additive covariate (Fig 1 and Table 2). When sex was included as an interactive covariate, the QTL on Chromosome 5 reached a LOD score of 4.77 (P < 0.1) and the ΔLOD was ≥ 2.0; therefore male and female cohorts were analyzed separately. In females, a significant QTL was localized to Chromosome 5 and a suggestive QTL identified on Chromosome 12. In the male cohort, suggestive QTL were identified on Chromosomes 1 and 9 (Fig 1 and Table 2). Interestingly, for QTL on Chromosomes 1, 6, 9, and 12 the allele conferring the higher exercise time came from the NZW strain.

**Table 2:** Significant and suggestive QTL for pre-training, post-training, and change in exercise time in NZW/LacJ x 129S1/SvImJ F_2_ mice.

Trait	Chr	Position, cM	95% CI,cM	LOD	High Allele	Nearest Marker
Pre-training	1	46.08	36–112	2.59 ^(M)^	NZW	rs3022821
	5	37.43	27–67	4.26* ^(F)^	S1	rs3715307
	5	57.37	11–69	2.71	S1	rs3668084
	6	47.81	24–62	2.93	NZW	rs3715132
	9	21.19	2–30	2.60 ^(M)^	NZW	rs3023203
	12	72.06	54–88	2.26 ^(F)^	NZW	rs3719660
Post-training	1	55.73	38–76	2.30	NZW	rs3684654
	5	43.43	35–56	4.14*	S1	rs3705373
	5	43.43	11–81	2.68^(M)^	S1	rs3705373
	8	66.12	2–90	2.22	S1	rs3726020
	8	84.18	2–80	2.43 ^(F)^	S1	rs4227443
	9	64.38	50–83	2.47 ^(F)^	S1	rs3687598
	14	19.63	10–28	2.71 ^(F)^	S1	rs3671357
Delta	1	21.13	8–72	2.47 ^(F)^	S1	rs4222256
	6	35.66	18–58	2.43 ^(F)^	S1	rs3655236
	6	37.81	14–62	2.73	S1	rs3655236

Chr, chromosome; 95% CI, 95% confidence interval in centimorgans (cM), CI was calculated using Bayesian credible interval; LOD, peak LOD score obtained in interval mapping using sex as an additive covariate; High Allele, allele with the highest exercise time; Nearest Marker, SNP marker closest the LOD peak; Delta, change in exercise time (post minus pre);

^(F)^, LOD score for QTL identified in female mice;

^(M)^, LOD score for QTL identified in male mice;

*, P < 0.05.

**Fig 2:**
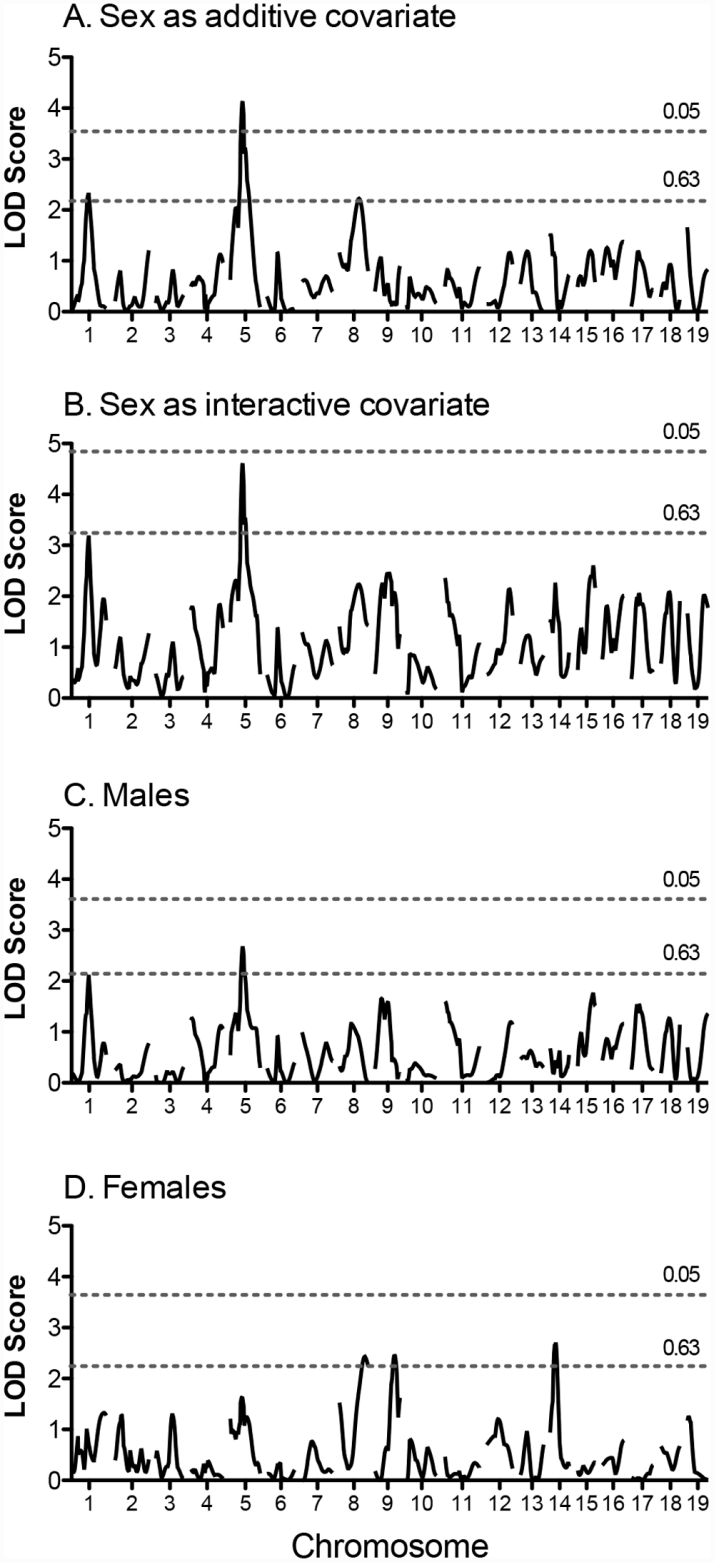
Genome-wide scan for post-training exercise time in (NZW x 129S1) F_2_ mice. Scans were performed for the entire population with “sex” as an additive covariate (A) or interactive covariate (B), and in males (C) and females (D) separately. Horizontal lines represent significant (P = 0.05) and suggestive (P = 0.63) logarithm of odds (LOD) thresholds, respectively. LOD thresholds were determined by permutation testing using 1000 permutations.

Fig 2 shows genome-wide linkage scans for post-training exercise time with sex as a covariate and in male and female cohorts. A significant QTL for post-training time was identified on Chromosome 5 in the entire cohort (Fig 2 and Table 2). Suggestive QTL were detected on Chromosomes 1 and 8 in this population. In the female only cohort, suggestive QTL were identified for post-training time on Chromosomes 8, 9, and 14. One suggestive QTL, which overlapped with the significant QTL on Chromosome 5, was identified in the male only cohort. For the significant QTL on Chromosome 5, the 129S1 allele conferred the greater exercise time and this QTL explained 3.7% of the phenotypic variance. The 95% confidence interval for this significant QTL overlaps with the suggestive QTL for pre-training time.

**Fig 3:**
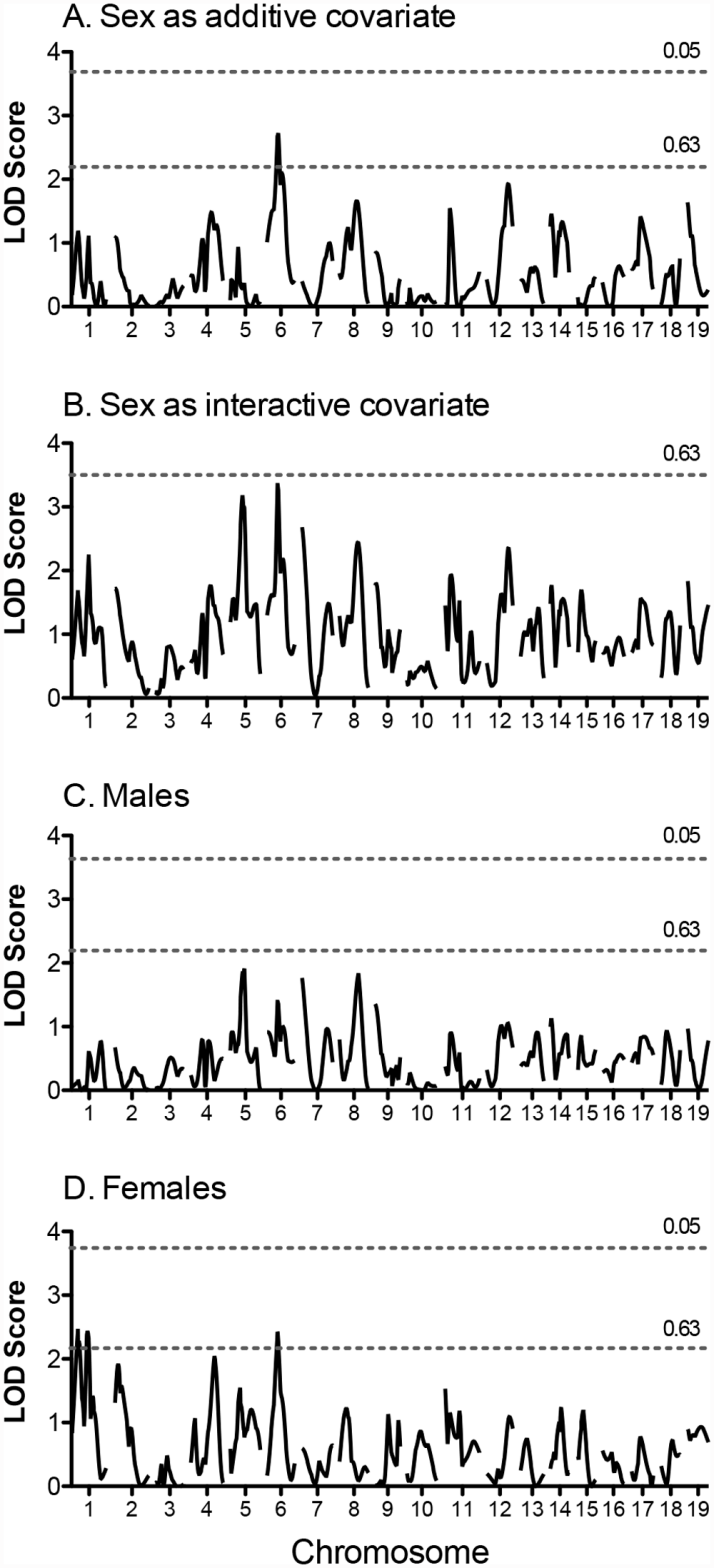
Genome-wide scan for the change exercise time in response to training in (NZW x 129S1) F_2_ mice. Scans were performed for the entire population with “sex” as an additive covariate (A) or interactive covariate (B), and in males (C) and females (D) separately. Horizontal lines represent significant (P = 0.05) and suggestive (P = 0.63) logarithm of odds (LOD) thresholds, respectively. LOD thresholds were determined by permutation testing using 1000 permutations.

Genome-wide linkage scans for the responses to exercise training (i.e., change in exercise time) are shown in Fig 3. One suggestive QTL was identified for the response to training on Chromosome 6 in the entire cohort and two suggestive QTL were detected in the female only group for this phenotype. The 95% confidence interval for the suggestive QTL for the response to training on Chromosome 6 overlaps with the suggestive QTL for pre-training time on this chromosome.

### Combined cross genome-wide linkage analysis

**Fig 4:**
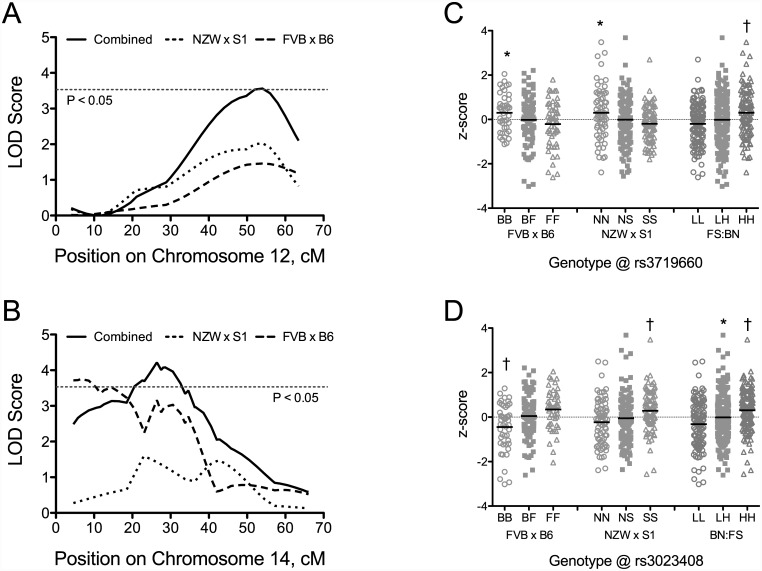
Novel QTL for pre-training exercise time identified on Chromosomes 12 and 14 using combined cross analysis. LOD score plots for pre-training exercise time on Chromosome 12 (A) and 14 (C) are shown for FVB x B6 F_2_ (dashed line), NZW x 129S1 F_2_ (dotted line) and combined cross (sold line). Horizontal dashed line represents significant (P = 0.05) LOD threshold for the combined cross. Allele effect plots for QTL for pre-training exercise time on Chromosomes 12 (B) and 14 (D). The y-axis in each graph is the z-score transformed exercise time and the x-axis indicates cross-specific genotypes. Homozygous S1, NZW, FVB, and B6 are denoted “SS”, “NN”, “FF”, and “BB”, respectively. Heterozygous alleles are denoted “NS” and “BF” in individual crosses. For combined cross analysis, genotypes were re-coded as “HH” for high performing strains and “LL” for low performing strains, and “LH” for heterozygotes. The strain pattern for high and low performing strains is indicated below allele effect plot for each combined cross QTL. Allele effects are shown at the peak location for each QTL. The solid black line for each genotype represents the mean. *, P < 0.05 compared with alternate homozygous genotype (e.g., BB vs. FF); †, P < 0.05 compared with other genotypes.

Combined cross analysis was performed for pre- and post-training exercise time and the response to training (S1 and S2 Figs). In combining crosses, 9 suggestive or significant QTL were identified for pre-training time (Table 3). Seven of these QTL were identified as suggestive or significant in the individual crosses. Two QTL for pre-training time, identified on Chromosomes 11 and 12 in the combined cross (Table 3), were not identified in the individual crosses. The LOD score and effect plots for the individual and combined crosses for the significant QTL on Chromosome 12 are shown in Fig 4A and 4B. For this QTL, the B6 and NZW alleles were associated with higher exercise times and in individual crosses displayed an additive pattern of inheritance. The significant QTL for pre-training time on Chromosome 14 is shown in Fig 4C and 4D. This QTL was identified in the individual FVB x B6 F_2_ intercross. The LOD score in the combined cross is slightly higher (4.2 vs. 3.7) and the 95% CI is smaller (26 cM vs 28 cM) than for the individual FVB x B6 cross. For this QTL, homozygous B6 genotype (BB) was associated with a lower exercise time compared with the other genotypes (Fig 4D). Similarly, for the NZW x 129S1 intercross, homozygous “SS” mice had significantly higher exercise time than either heterozygous “NS” or homozygous “NN” genotypes.

**Table 3:** Significant and suggestive QTL for exercise time and the response to training identified using combined cross analysis.

	FVB x B6 F_2_		NZW x 129S1 F_2_		Combined Crosses ^a^		
Chr	Position, cM (95% CI)	LOD	Position, cM (95% CI)	LOD	Position, cM (95% CI)	LOD	Strain Pattern(LL:HH)
Pre-training time							
2	52 (32–66)	3.3†	103 (8–103)	1.3	50 (36–103)	2.4	BS:FN
3	53 (44–79)	2.2§	50 (2–79)	0.6	53 (2–79)	2.3	FN:BS
5	48 (3–89)	1.0	56 (11–65)	2.7§	56 (31–65)	3.2†	FN:BS
6	26 (4–78)	0.6	36 (16–50)	2.9§	30 (12–46)	2.5	FS:BN
9	21 (6–26)	2.4§	6 (2–73)	1.2	16 (2–26)	2.6	BS:FN
11	38 (21–85)	1.6	22 (3–79)	1.4	22 (3–65)	2.2	BN:FS
12	54 (26–63)	1.5	54 (30–63)	2.0	54 (42–60)	3.6*	FS:BN
14	7 (5–33)	3.8*	23 (10–53)	1.6	26 (11–37)	4.2*	BN:FS
19	34 (20–50)	3.8*	34 (6–57)	0.7	36 (18–50)	3.1	FN:BS
Post-training time							
3	54 (46–76)	3.6*	50 (2–79)	0.8	50 (44–78)	3.0	FS:BN
5	89 (3–89)	1.3	39 (33–53)	4.1*	33 (6–61)	2.8	BN:FS
8	60 (2–72)	2.3§	52 (2–68)	2.2§	60 (50–70)	3.1	FN:BS
14	34 (26–39)	5.1*	9 (5–66)	1.5	36 (5–63)	2.8	BS:FN
19	52 (42–57)	2.6§	4 (4–57)	1.7	52 (42–57)	2.7	FN:BS
Change in time							
6	48 (22–78)	1.7	28 (10–50)	2.7§	28 (20–52)	3.7*	FN:BS
8	64 (2–72)	1.3	46 (12–62)	1.7	45 (38–62)	2.8	FN:BS
11	39 (29–49)	3.7*	22 (9–85)	1.5	25 (19–77)	2.4	FS:BN
19	26 (4–57)	2.1	4 (4–57)	1.6	22 (6–32)	2.4	BS:FN

Chr, chromosome; LOD, logarithm of odds; Position, position of QTL peak (and 95% confidence interval) in cM from the analysis using cross and sex as additive covariates.

^a^, All combined cross QTL peaks have surpassed suggestive (P < 0.63) threshold;

*, P < 0.05;

^†^, P < 0.10;

^§^, P < 0.63 in individual F_2_ intercrosses

**Fig 5:**
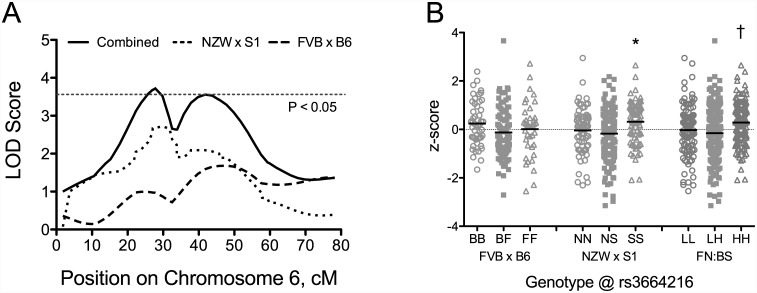
LOD score plots (A) and allele effect plots (B) for QTL for change in exercise time on Chromosome 6 using combined cross analysis. LOD score plots are shown for FVB x B6 F_2_ (dashed line), NZW x 129S1 F_2_ (dotted line) and combined cross (sold line). Horizontal dashed line represents significant (P = 0.05) LOD threshold for the combined cross. For allele effect plots, the y-axis is the z-score transformed change in exercise time and the x-axis indicates cross-specific genotypes. Homozygous S1, NZW, FVB, and B6 are denoted “SS”, “NN”, “FF”, and “BB”, respectively. Heterozygous alleles are denoted “NS” and “BF” in individual crosses. For combined cross analysis, genotypes were re-coded as “HH” for high performing strains (B6, 129S1) and “LL” for low performing strains (FVB, NZW), and “LH” for heterozygotes. Allele effects are shown at the peak location for the QTL. The solid black line for each genotype represents the mean. *, P < 0.05 compared with alternate homozygous “NN” genotype; †, P < 0.05 compared with other genotypes.

For post-training exercise time, 5 suggestive QTL were identified using combined cross analysis (Table 3). Each of these QTL were identified in at least one individual cross and only the QTL on Chromosome 8 was identified as suggestive in both crosses. Four suggestive or significant QTL were identified for the response to training (change in exercise time) after combining crosses. The suggestive QTL on Chromosomes 8 and 19 were not present in individual crosses, whereas the suggestive QTL on Chromosome 11 was identified in the FVB x B6 intercross. The significant QTL on Chromosome 6 was identified as a suggestive QTL in the current NZW x 129S1 intercross (Table 2). After combining crosses, the LOD score increased to 3.7 (P < 0.05) and the peak of the QTL was located at 28 cM (Fig 5A). The LOD score plot for the combined cross shown in Fig 5A suggests the presence to two QTL on Chromosome 6; however, the evidence for two additive QTL at 27 cM and 46 cM did not reach significance (lod.add = 5.04, P = 0.001; lod.av1 = 1.36, P = 0.198). These positions are however close to the positions of the highest LOD scores obtained on Chromosome 6 in the individual crosses. The allele effect plots for the significant QTL on Chromosome 6 is shown in Fig 5B. Mice homozygous for 129S1 allele had significantly greater change in exercise time than mice homozygous for the NZW allele. There were no significant differences among genotypes in the FVB x B6 intercross, although mice homozygous for the B6 allele showed slightly greater responses to training than mice with alternative genotypes. Therefore, in the combined cross analysis 129S1 and B6 alleles were coded as high and NZW and FVB alleles coded as low, which resulted in homozygous “HH” mice having significantly greater responses to training than homozygous “LL” or heterozygous “LH” mice (Fig 5B).

## Discussion

Genetic factors determining exercise capacity and the magnitude of the response to exercise training are poorly understood. The aim of this study was to identify QTL for exercise capacity and training responses in an independent inbred line cross and to use combined cross analysis to improve localization of shared QTL. Based on marked differences in training responses in inbred NZW and 129S1 mice, genome-wide linkage scans were performed for pre-training and post-training exercise time, and for the change in exercise time in F_2_ mice derived from these strains. Suggestive or significant QTL were identified for pre-training exercise time (Chrs 1, 5, 6, 9, and 12), post-training exercise time (Chrs. 1, 5, 8, 9, and 14) and the change in exercise time (Chrs 1 and 6). To identify shared QTL, this data set was combined with data from a previously published cross between B6 and FVB strains. In the combined cross analysis, we identified new QTL for exercise capacity and responses to training not evident in individual crosses. Notably, a significant QTL for pre-training exercise time was identified on Chromosome 12 (54 cM, 3.6 LOD) and a significant QTL for the response to training was identified on Chromosome 6 (28 cM, 3.7 LOD). In addition, confidence intervals for QTL identified in Chrs 5, 8, and 14 in individual crosses were reduced using combined cross analysis. These results support the use of combining data from multiple crosses to identify QTL for exercise-related traits. The number of novel QTL identified in the current study underscore the complexity of exercise capacity and training responses as heritable traits and confirm that additional genetic information from multiple approaches is required to elucidate causal genes and/or genetic variants underlying exercise capacity and training responses.

This study expands the current knowledge about exercise capacity and responses to training in inbred mice as well as the genetic loci regulating these phenotypes. The new intercross in the current study involved NZW and 129S1 strains. These strains were identified in our laboratory as having significantly different baseline or pre-training exercise capacity [19] and responses to training (Table 1). Interestingly, the NZW mice showed no response to 4 weeks of treadmill running at ~65% of maximum. Mice from the 129S1 strain showed an approximately 21% increase in exercise time, providing evidence that the protocol was sufficient to elicit a response. The lack of response to training is well documented in rodents and humans. We previously reported that mice from inbred and hybrid strains failed to significantly increase exercise capacity in response to training [28]. Koch et al. also reported that inbred Copenhagen rats exhibited a low baseline (intrinsic) exercise capacity and no or little response to 8 weeks of treadmill running at either an absolute or relative workload, whereas rats from the Dark Agouti strain improved their exercise capacity by 50% and 36%, respectively in response to these training regimens [39]. In humans, multiple families showed a negative or zero response to training in the HERITAGE study [7], and similar observations have been reported in other exercise training studies [8–11]. Collectively, these data provide evidence for a nonresponse to training across species and support the idea that different genetic factors determine baseline exercise capacity and the response to training.

In the NZW x 129S1 cross, suggestive QTL for pre-training exercise time were identified on Chrs 5 and 6 (Fig 1, Table 2). These QTL have not been identified previously in rodents, suggesting that they are novel QTL contributing to the genetic regulation of exercise capacity in mice. As reported by us, and others, pre-training exercise capacity differs significantly between males and females, therefore the influence of sex on QTL for pre-training exercise time was considered. Using sex as a covariate, 4 QTL were identified in either male or female populations of the NZW x 129S1 cross, with only the QTL on Chr 5 identified in the female cohort overlapping with a QTL identified in the entire cohort. In the female cohort, the QTL on Chr 5 surpassed the significance threshold, whereas in the entire cohort, the LOD score only reached the suggestive level, implying that this might be a female-specific QTL. Sex-specific QTL have been reported for exercise capacity [17, 21] and related traits such as physical activity measured by wheel running [40, 41]. In fact, most of QTL identified in a genome-wide association study of voluntary wheel running were identified in one sex only [40]. Therefore the influence of sex on exercise capacity and related traits should be taken into consideration when studying the genetic architecture of this trait.

Nine QTL were identified for pre-training exercise time using combined cross analysis. The majority of these QTL are considered suggestive and were identified in at least one individual cross (Table 3). However, combined cross analysis narrowed the 95% CIs of the QTLs on Chrs 5 and 14 identified in individual crosses. The significant QTL on Chr 14 is concordant with regions of the human genome identified in the HERITAGE Family Study linked to mean power output, maximal oxygen consumption in the sedentary state (VO_2max_), and changes in VO_2max_ with training (ΔVO_2max_) [12, 13]. In addition, SNPs associated with training responses at maximal (ΔVO_2max_) and submaximal workloads (ΔVO_2_) in humans also map to regions sytenic with the QTL on Chr 14 [15, 42]. Similarly, genes with genetic markers or SNPs associated with power output during exercise (PLCE1) [13], training responses (BTAF1, PIP5K1B) [15, 43], and fitness (ANKRD22) [44] in humans are located in the combined cross QTL on Chr 19 (Table 3). Each of these genes has a mouse homolog, *Plce1*, *Btaf1*, *Pip5k1b*, and *Ankrd22*, respectively (http://www.informatics.jax.org/homology.shtml). Of these genes, *Plce1* (phospholipase C, epsilon 1) contains multiple SNPs that match the combined cross analysis strain distribution pattern for this QTL (FN:BS); however, each of these SNPs falls within intronic regions of *Plce1*. Similarly, *Pip5k1b* (phosphatidylinositol-4-phosphate 5-kinase, type 1 beta) contains 175 intronic SNPs that match the strain distribution pattern for this QTL. Thus, at least two genes identified in human studies also were identified in exercise-related QTL in the mouse and contain SNPs matching the expected strain distribution pattern. However, their contribution to variation in pre-training exercise capacity is not clear.

The other significant QTL for pre-training exercise time identified using the combined cross analysis is located on Chr 12. This QTL was not identified in either of the individual crosses, indicating that this a novel QTL for pre-training exercise time. The CI for this QTL does not overlap with any known QTL for exercise capacity or responses to exercise training. Overall, 247 genes map to the 95% CI of this QTL (~21 Mb). Ten of these genes contain polymorphisms that match the strain distribution patterns for this QTL and result in a change in amino acid sequence (coding nonsynonymous). One of these genes, tyrosyl-DNA phosphodiesterase 1 (*Tdp1*), is a mitochondrial enzyme involved in mitochondrial DNA repair [45]. A mutation in TDP1 in humans has been linked to spinocerebellar ataxia [46], but this was not recapitulated in the mouse [47]. Several genes belong to the *Serpin* family of genes, including isoforms of *Serpina3 (a-c*, *f-n)* and *Serpina1 (c-f)*. The mouse genome contains six *Serpina1* genes (a-f) and nine *Serpina3* genes (a-c, f-n) [48]. In humans, SERPINA3, also known as alpha-1-antichymotrypsin, is thought to be part of the inflammatory responses to exercise [49, 50]. This gene has four homologs in the mouse, *Serpina3c*, -*k*, -*m*, and -*n* (http://www.informatics.jax.org/homology.shtml), none of which have been linked to exercise performance. Although the isoform was not specified, Chaillou et al. recently reported that *Serpina3* is upregulated in mouse plantaris muscle during periods of muscle hypertrophy and regrowth [51]. Limited data also suggests that there may be strain differences in expression of *Serpina3i* in both adipose tissue and liver that match the strain distribution pattern at the QTL peak SNP [52, 53]. However, this remains to be confirmed. In humans, SERPINA1 or alpha-1-antitrypsin plays a role in inhibition of neutrophil proteases and deficiency of SERPINA1 is linked to emphysema [54]. This gene also has been related to the anti-inflammatory effects of exercise [55]. SERPINA1 has five homologs in the mouse: *Serpina1a-e* (http://www.informatics.jax.org/homology.shtml). Four of these genes are in our QTL interval, but their role in exercise capacity and responses to training are not clear. Overall, these data provide evidence that this region on Chr 12 could house genes that regulate variation in baseline exercise capacity; however, additional research is required to confirm the importance of this region on baseline exercise capacity.

Six suggestive and significant QTL were identified for post-training exercise time in the NZW x 129S1 cross. Only two QTL overlap with QTL identified for pre-training exercise capacity, suggesting that the genetic architecture for these two traits are somewhat different. Several of these QTL (Chrs 1, 9, and 14) overlap with previously identified QTL in humans. For example, the QTL on Chr 1 overlaps with syntenic regions in humans that contain QTL for mean power output, VO_2max_, and ΔVO_2max_ [12, 13]. Two subsequent studies identified genomic features associated with training responses that also map to this region on Chr 1 [14, 44], suggesting that Chromosome 1 should be considered for more detailed analyses of the genetic basis for exercise training responses. In the combined cross analysis, 5 suggestive QTL were identified for post-training exercise time. All of these QTL were identified in one of the individual crosses. The QTL on Chr 8 was identified in both crosses. Although the LOD score for this QTL was not markedly higher in the combined cross analysis, the 95% CI was considerably smaller (~20 cM vs. ~70 cM). A marker linked to VO_2max_ in the sedentary state in humans maps to a region syntenic to the post-training QTL on Chr 8 [13]. A suggestive QTL on Chr 14 was also identified in the combined cross. This QTL overlaps with a QTL for post-training time identified in the female cohort of the NZW x 129S1 cross and a significant QTL for post-training work previously identified in the FVB x B6 cross. This region contains the gene mitochondrial intermediate peptidase (*Mipep*). In humans, MIPEP is highly expressed in heart and skeletal muscle and thought to play a role in neurodegenerative disease [56, 57]. A SNP in this gene was one of several used to predict responses to exercise training based on baseline gene expression in skeletal muscle [15]. The QTL on Chr 5 also overlaps with sytenic regions in humans for exercise performance and training-related traits identified in the HERITAGE Family Study [12, 13].

Three suggestive QTL for the response to training (change in time) were identified in the NZW x 129S1 cross. One QTL on Chr 6 was identified using the full F_2_ population and subsequent analysis in sex specific populations identified a similar QTL in the female only cohort, suggesting that this might be a female-specific QTL. The QTL on Chr 1 was also only identified in females. Identifying these QTL in females only is somewhat surprising given there were no significant differences in the response to training between male and female F_2_ mice in this cross. However, it is clear from this study and others that genetic regulation of many complex traits can differ between males and females, regardless of the level of phenotypic differences [17, 20, 21, 36, 40, 41, 58, 59].

In the combined cross analysis, a significant QTL for the change in time with training also was identified on Chr 6. The 95% CI for this QTL overlaps with the QTL identified for the same trait in the NZW x 129S1 cross, and was not identified in the FVB x B6 cross. Although the shape of the LOD curve in the combined analysis suggests the possibility of two closely linked QTL (Fig 5), results from the pair scan failed to provide strong evidence for the presence of two QTL on this chromosome. This QTL overlaps with several QTL identified in humans and contains a carboxypeptidase gene, CPVL, a transcript included in a group of genomic predictors for responses to exercise training in humans [15]. In the mouse homolog, *Cpvl*, several SNPs match the strain distribution pattern for this QTL, but all are intronic. A suggestive QTL on Chr 19 was also identified in the combined cross analysis. This QTL overlaps with the QTL for pre-training exercise time (Table 3), and contains genes associated with training responses (BTAF1, PIP5K1B), and fitness (ANKRD22) in humans as described above.

## Conclusions

Because the number of inbred strains crosses for exercise capacity or training responses is small, the ability to identify additional QTL and potential candidate genes using combined cross analyses is limited. However, combining data from two inbred strain crosses, novel QTL not present in individual crosses were identified. These results support the utility of using larger and more diverse mapping populations to identify the genetic basis for exercise capacity and responses to training. Future studies focusing on larger intercross populations, a large number of strains (e.g., hybrid mouse diversity panel) or more genetically diverse populations (e.g., Collaborative Cross or heterozygous stock mice or rats) would likely lead to discovery of more small effect QTL. In addition, several of the QTL identified in the NZW x 129S1 intercross were present in single sex, primarily female only, cohorts. These results provide supportive evidence that sex differences in exercise capacity and responses to training are, in part, due to genetic differences and future studies should incorporate animals of both sexes for genetic analyses. Finally, many of the QTL identified in individual and combined crosses were potentially concordant with exercise and exercise training-related QTL in humans. The concordance supports the importance of these regions in determining the genetic basis for variation in exercise capacity and responses to training. Although no strong candidate genes were identified in this study, those genes identified in both human and mouse studies warrant further investigation as possible candidates for genetic regulators of exercise capacity and responses to training.

## Supporting Information

S1 Fig Genome-wide scans for pre-training (A), post-training (B), and change in exercise time (C) in combined crosses. Scans were performed on z-score transformed phenotypes for the entire population with “sex” and “cross” as additive covariates. FVB and 129S1 strains were coded as high and B6 and NZW strains as low. Horizontal lines represent significant (P = 0.05) and suggestive (P = 0.63) logarithm of odds (LOD) thresholds, respectively. LOD thresholds were determined by permutation testing using 1000 permutations.
(TIFF)

S2 Fig Genome-wide scans for pre-training (A), post-training (B), and change in exercise time (C) after re-coding combined crosses. Scans were performed on z-score transformed phenotypes for the entire population with “sex” and “cross” as additive covariates. FVB and NZW strains were coded as low and B6 and 129S1 strains as high. Horizontal lines represent significant (P = 0.05) and suggestive (P = 0.63) logarithm of odds (LOD) thresholds, respectively. LOD thresholds were determined by permutation testing using 1000 permutations.
(TIFF)
