# Supplementary figures and images for: Exercise Capacity and Response to Training Quantitative Trait Loci in a NZW X 129S1 Intercross and Combined Cross Analysis of Inbred Mouse Strains
Source: PLoS One. 2015 Dec 28;10(12):e0145741. doi: 10.1371/journal.pone.0145741 (PMC4692404; doi:10.1371/journal.pone.0145741)

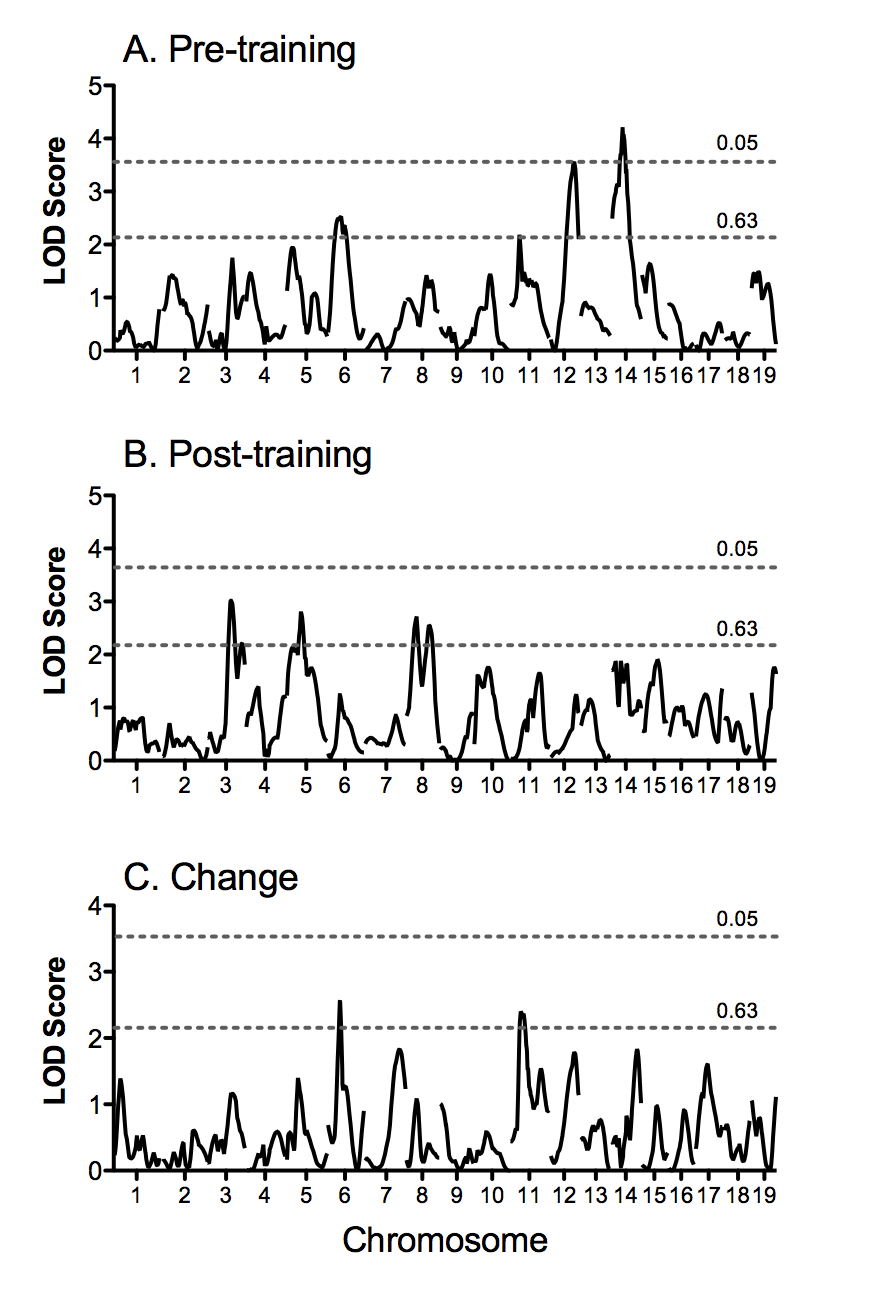

Supplement: S1 Fig — Scans were performed on z-score transformed phenotypes for the entire population with “sex” and “cross” as additive covariates. FVB and 129S1 strains were coded as high and B6 and NZW strains as low. Horizontal lines represent significant (P = 0.05) and suggestive (P = 0.63) logarithm of odds (LOD) thresholds, respectively. LOD thresholds were determined by permutation testing using 1000 permutations. (TIFF) [file pone.0145741.s001.tiff]

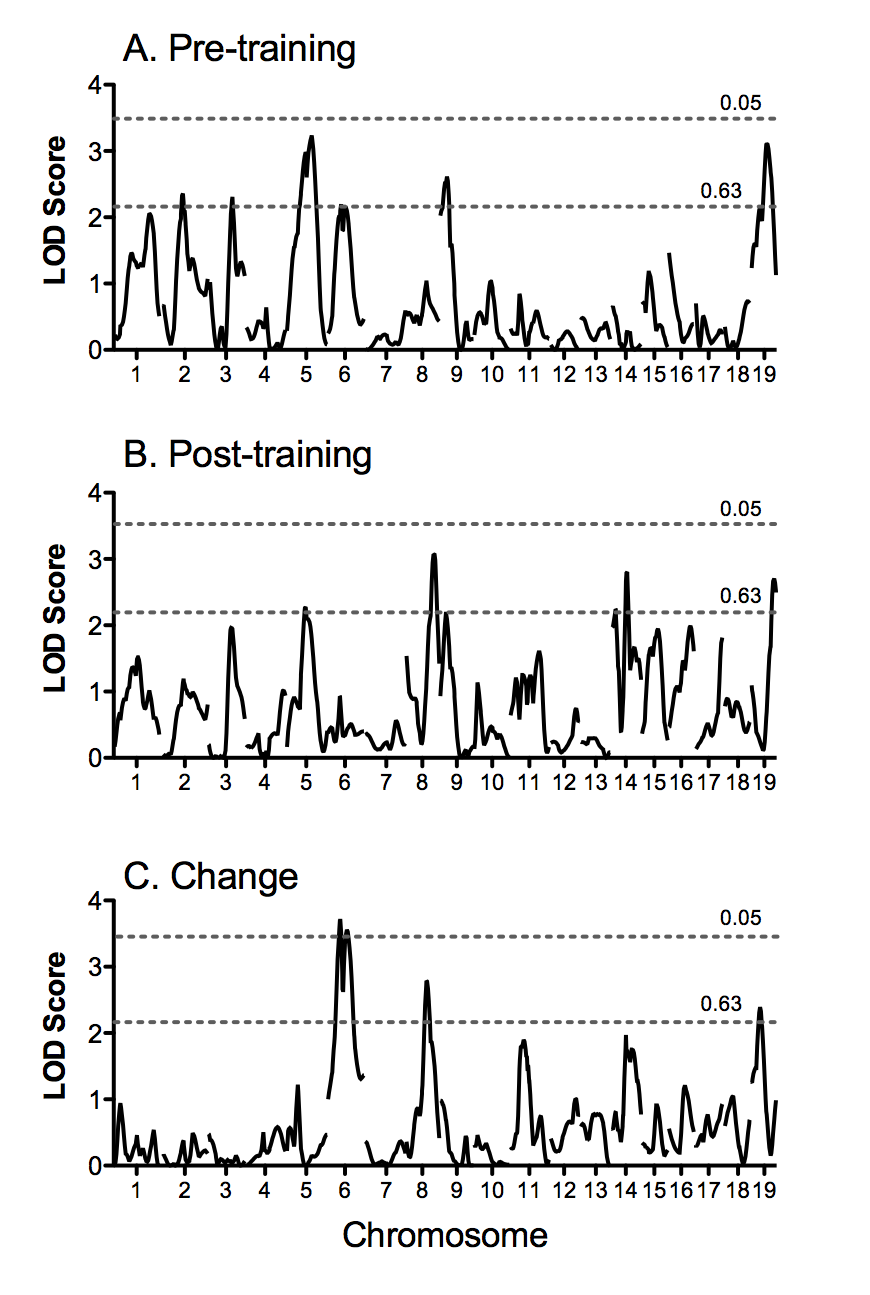

Supplement: S2 Fig — Scans were performed on z-score transformed phenotypes for the entire population with “sex” and “cross” as additive covariates. FVB and NZW strains were coded as low and B6 and 129S1 strains as high. Horizontal lines represent significant (P = 0.05) and suggestive (P = 0.63) logarithm of odds (LOD) thresholds, respectively. LOD thresholds were determined by permutation testing using 1000 permutations. (TIFF) [file pone.0145741.s002.tiff]
